# The Incorporation of Strontium in a Sodium Alginate Coating on Titanium Surfaces for Improved Biological Properties

**DOI:** 10.1155/2017/9867819

**Published:** 2017-10-03

**Authors:** Ning Yuan, Lili Jia, Zhen Geng, Renfeng Wang, Zhaoyang Li, Xianjin Yang, Zhenduo Cui, Shengli Zhu, Yanqin Liang, Yunde Liu

**Affiliations:** ^1^Department of Laboratory Medicine, Tianjin Chest Hospital, Tianjin 300051, China; ^2^School of Laboratory Medicine, Tianjin Medical University, Tianjin 300072, China; ^3^School of Materials Science & Engineering, Tianjin Key Laboratory of Composite and Functional Materials, Tianjin University, Tianjin 300350, China

## Abstract

Orthopedic implant failure is mainly attributed to the poor bonding of the implant to bone tissue. An effective approach to minimize the implant failure would be modifying the surface of the implant. Strontium (Sr) can stimulate the proliferation and differentiation of osteoblasts and reduce the activity of osteoclasts. In this study, a titanium (Ti) surface was successively functionalized by covalently grafting dopamine, sodium alginate (SA), and Sr^2+^ via the electrostatic immobilization method. The as-prepared coatings on the Ti surface were characterized by using scanning electron microscopy (SEM), X-ray photoelectron spectroscopy (XPS), Fourier transform infrared spectroscopy (FT-IR), and contact angle. The results indicated that the Sr-incorporated coatings were successfully prepared and that Sr distributed uniformly on the surface. A long-lasting and sustained Sr release had been observed in Sr^2+^ release studies. The Ti/DOPA/SA/Sr exhibited little cytotoxicity and a robust effect of Sr incorporation on the adhesion and spreading of MG63 cells. The proliferation and alkaline phosphatase (ALP) activity of MG63 cells were enhanced by immobilizing Sr^2+^ on the SA-grafted Ti. The Sr-containing coatings, which displayed excellent biocompatibility and osteogenic activity, may provide a promising solution for promoting the tissue integration of implants.

## 1. Introduction

Titanium (Ti) and its alloys, which have excellent mechanical properties and chemical stability, are commonly used as orthopedic and dental implant materials [1–3]. A spontaneously formed oxide layer on a Ti surface can provide it with biocompatibility and bioactivity [4]. On attaining complete adhesion between the implant and host bone tissue, however, it is not efficient in terms of the risk of the implant loosening over time, resulting in ultimate implant failure [5]. Consequently, many efforts are being devoted to methods of modifying the surface of Ti to achieve the desired biological responses [6]. The titanium oxide layer with an incorporated bioactive ion, which promotes osteoblast differentiation and increases biomechanical anchorage, has become a very active research area [7–10].

Strontium (Sr), which has recently drawn a lot of attention, plays an important role in the enhancement of implant bone healing. Several researchers involved with in vivo and in vitro studies have reported the beneficial effects of Sr on bone metabolism [11–13]. In vivo, Sr presents in the region of bone mineralization. It can stimulate the proliferation and differentiation of osteoblasts and inhibit the activity of osteoclasts, consequently enhancing matrix deposition and ultimately new bone formation [14]. Strontium ranelate has been proven to favor bone healing by both increasing new bone formation and reducing bone resorption [15–18].

In recent studies, Sr has been experimentally incorporated into various bone implantation biomaterials [13, 16, 17, 19–23]. Sr incorporations in calcium phosphate and hydroxyapatite coatings have been widely investigated. Li et al. [17] found that Sr^2+^-substituted hydroxyapatite coatings with 10 mol% Ca^2+^ replaced by Sr^2+^ could enhance implant osseointegration in ovariectomized rats. Capuccini et al. [16] showed that hydroxyapatite substituted by a suitable dosage of strontium could promote the attachment and proliferation of osteoblasts. Ni et al. [13] demonstrated that Sr-containing hydroxyapatite bone cement exhibited better bioactivity than hydroxyapatite bone cement in a revision hip replacement model using goats. Thus, Sr incorporation may be considered as an effective way to enhance the osteoconductivity of implants. It would also be expected that Sr-containing organic coatings on Ti might improve implant bone healing. Sodium alginate (SA), an organic polymer material with plenty of hydroxyl and carboxyl, has good chelation of Sr [24–26]. It could be an efficient delivery platform with controlled Sr releasing. Moreover, as an abundant natural polysaccharide, SA has excellent biocompatibility and stability [27]. Thus, the SA coating incorporated with Sr^2+^ on Ti is expected to have considerable potential in implant modification.

It is difficult to prepare the SA coating for Ti with a smooth surface. Inspired by the adhesive proteins secreted by mussels for attachment to wet surfaces, several researchers have found dopamine (DOPA, or 3,4-dihydroxy-phenylalanine) to be an appropriate main constituent to attach to the wet surface [28–30]. It could form strong covalent and noncovalent interactions with substrates. Moreover, the existence of catechol groups in DOPA makes it a potentially excellent anchor on all types of materials.

**Figure 1:**
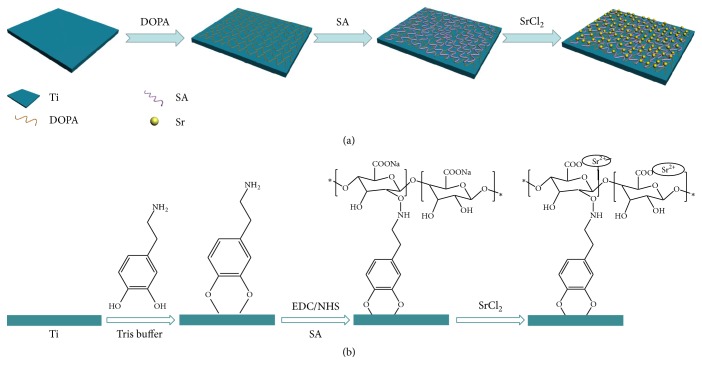
(a) Schematic illustration of the preparation process of Sr-incorporated alginate coating on titanium surface. (b) The molecular formula of Sr-incorporated alginate coating on titanium surface.

In this study, a SA coating-incorporated Sr^2+^ is fabricated via the strong interaction of DOPA on Ti. The experiment's schematic is shown in Figure 1. The composition and properties of the modified Ti surface are subsequently investigated and analyzed. Finally, cell morphology, cytotoxicity, proliferation, and ALP activity are assayed.

## 2. Materials and Methods

### 2.1. Materials

Commercial pure Ti (99.6%) was purchased from Zhongtian Co., Ltd. (China). Dopamine (dopamine hydrochloride), sodium alginate (Mw~25 kDa), hydrochloric acid, sodium hydroxide, acetone, ethyl alcohol, tris(hydroxymethyl)aminomethane, strontium chloride, 1-ethyl-(3-dimethylaminopropyl)carbodiimide (EDC), and N-Hydroxysuccinimide (NHS) were obtained from Sigma-Aldrich (USA).

### 2.2. Preparation and Pretreatment of Ti

The commercial pure Ti was cut into 10 mm × 15 mm plates. These plates were polished using 800- and 1500-grid sandpaper and then were cleaned ultrasonically for 10 min successively in acetone, ethanol, and water. To wipe out the deposited carbide and prepare a tight porous structure, Ti plates were put into 40 wt.% HNO_3_ for ultrasonic cleaning for 40 min. Then the acid-treated plates were rinsed thoroughly with deionized water, placed in the 5 mol/L NaOH at 90°C for 5 h, and subsequently cleaned ultrasonically in water for 30 min.

### 2.3. Preparation of the Coatings on Ti

To prepare the DOPA coating on the Ti, DOPA was firstly anchored to the surface of the Ti after acid and alkali treatment. The Ti plates were immersed in a 1 g/L aqueous solution of DOPA overnight in the dark, and then the plates were rinsed with ultrapure water to remove the unattached DOPA and dried under the dynamic nitrogen atmosphere. The resulting plates were denoted as Ti/DOPA.

To prepare the SA coating on the Ti, 0.5 g SA was added to 50 ml H_2_O. After the SA was dissolved adequately in the water, 0.3881 g (50 mmol/L) EDC and 0.2875 g (50 mmol/L) NHS were put into the SA solution. Then, the SA solution continued to be stirred overnight. The Ti/DOPA samples were placed into SA solution at 37°C. After 12 h, the plates were taken out and rinsed with ultrapure water to remove the unattached SA. The SA-treated plates were denoted as Ti/DOPA/SA.

The Ti/DOPA/SA samples were treated in a 20 mL aqueous solution of SrCl_2_ overnight at 37°C to prepare the Sr coating. The SrCl_2_ concentrations were 1 wt.% and 5 wt.%, respectively. As noted in the following discussion, they were denoted as Ti/DOPA/SA/Sr1 and Ti/DOPA/SA/Sr5, respectively. After the overnight reaction, the surfaces were rinsed by pipetting 1 mL of ultrapure water over the surface to remove the excess SrCl_2_, and the rinse solutions were collected and stored for further analysis.

### 2.4. Surface Characterization

The functional groups of the surface coatings were analyzed by attenuated total reflection infrared (ATR-IR) spectroscopy. The ATR-IR spectra of the coatings were recorded on the spectrometer using a single-reflection horizontal ATR accessory (Bruker Vertex 70). Each spectrum was collected in the range of 4000−400 cm^−1^ by cumulating 32 scans at a resolution of 4 cm^−1^. The surface morphologies of the coatings were conducted by field-emission scanning electron microscopy (S-4800, Hitachi, Japan). An accelerating voltage of 10 kV was chosen for a scanning electron microscopy (SEM) analysis, and micrographs were captured using secondary electrons collected with an in-lens detector. To elucidate the status of the elements, the samples were characterized by XPS using a PHL1600ESCA instrument equipped with a monochromatic Mg Ka X-ray source (*E* = 1253.6 eV) operated at 250 W. The analysis spot had a diameter of 200 *μ*m, and the detection angle relative to the substrate surface was 45. The dynamic contact angle analysis using double-distilled water was performed utilizing a JCD2000 drop shape analysis system (Powereach Co., Shanghai, China). The initial distilled water volume was 5 *μ*L, and the measurement was performed at ambient temperature. For each group, the mean value of the contact angle was calculated from 5 individual measurements.

### 2.5. Sr^2+^ Release Characterization

Sr^2+^ release behaviors from Ti/DOPA/SA/Sr1 and Ti/DOPA/SA/Sr5 were investigated by immersion in 5 mL of phosphate-buffered saline (PBS) at 37°C. Measurements were taken at 12 h, 1 d, 2 d, 3 d, 5 d, 7 d, 9 d, 13 d, 17 d, 21 d, and 28 d. The amount of the released Sr^2+^ was measured using inductive coupled plasma optical emission spectroscopy (ICP-OES, Optima 3000 DV (PerkinElmer)). The Sr^2+^ concentration was calculated based on the previous measurements. Ultimately, the Sr^2+^ release curve was expressed in a plot with the accumulated Sr^2+^ versus time.

### 2.6. Cell Experiments

#### 2.6.1. Cell Culture

Osteoblast-like cells (MG63) were cultured in a high-glucose DMEM medium supplemented with 10% fetal bovine serum (FBS) and 100 unit/mL penicillin. Cell cultures were maintained at 37°C under a humidified atmosphere of 5% CO_2_ in air, and the growth medium changed every 2 to 3 days. The cells were passaged every 2 days.

#### 2.6.2. Cell Morphology

The samples were placed into 12-microwell plate and seeded with MG63 cells at a density of 2 × 10^4^ cells/well. After the MG63 cells were diluted by the DMEM medium, a 50 *μ*L cell suspension solution (2 × 10^4^ cells/50 *μ*L) was first carefully seeded on the samples, and then 1 mL medium was added to each sample well. After 6 h and 12 h of culturing, the medium was extracted, and 1 mL paraformaldehyde (4%) was put into each sample well to fix cells on the samples for 30 min. After this, 30%, 50%, 70%, 90%, and 100% ethanol aqueous solutions were successively added to the wells for cell dehydration. The treated samples were observed under SEM after being sputter-coated with gold.

#### 2.6.3. Cell Cytotoxicity Test

MG63 cells were seeded at a density of 4 × 10^4^ cells/well in a 12-well plate. The culture times of 1 d, 3 d, and 5 d were chosen to observe the toxicity of samples. After being cultured for 1 d, 3 d, and 5 d in a humidified atmosphere of 5% CO_2_, 10 *μ*L MTT (5 g/L) was added to every well, and the cells continued to incubate for 4 h at 37°C. After this, 1 mL DMSO was added to each well, and the 12-well plate was continuously oscillated for 10 min at 150 rpm. Then, the solution was transferred to a 96-well plate, and the absorbance was measured at 490 nm using a bioassay reader (HTS 7000 Plus, PerkinElmer Co., USA).

#### 2.6.4. Cell Adhesion, Distribution, and Proliferation

The cell adhesion and distribution of all the prepared samples were evaluated by representative fluorescence microscopy images of MG63 cells. The samples were placed into a 12-microwell plate and seeded with MG63 cells at a density of 1 × 10^4^ cells/well. After 1 day of culture, the medium was extracted, and the samples were rinsed with PBS 3 times; then, 500 *μ*L paraformaldehyde (4%) was put into each well to fix the cells on the samples for 30 min. After extracting the paraformaldehyde, the attached cells were stained by fluorescein isothiocyanate (FITC, Sigma), which highlights the cytoskeletal protein (excitation max.: 490 nm; emission max.: 520 nm; Sigma, USA; 5 *μ*g/ml of DMSO). This dye was applied for 30 min at room temperature. The microscopy images were acquired using an IX-51 microscope equipped with a digital camera (DP-70, Olympus, Japan).

After seeding for 1, 3, and 7 days, the MG63 cells on different samples were detached using a trypsin-EDTA solution (Sigma, USA, cat. number T4174) in PBS for 10 min at room temperature, and the number of cells was evaluated using a Vi-CELL XR analyzer (Beckman Coulter, USA).

#### 2.6.5. Alkaline Phosphatase Activity

The MG63 cells were seeded onto samples at a density of 1 × 10^4^ cells/well on a 12-microwell plate. After 3, 7, and 14 days of culture, the cultural medium was carefully extracted, and the coatings were gently washed twice with PBS. Then, 500 *μ*L of 0.2% (v/v) Triton X-100 (Sigma, USA) was added to each well for lysis for 2 h. After the solutions became homogeneous, 3 mL Coomassie Brilliant Blue staining solution, 0.6 mL cell lysis solution, and 0.4 mL double distilled water were added and mixed for 10 min, and the OD value of the mixed solution was measured using an ELISA reader (Tecan, Austria) at 595 nm. The protein concentration was determined with bovine serum albumin as a standard. Then, 100 *μ*L of the cell lysis solution and 100 *μ*L of 25 *μ*g/mL p-nitrophenyl phosphate disodium salt were added to each well of a 96-well plate (6 wells in each group), after which the reaction was stopped by adding 50 *μ*L NaOH (3 mol/L), 100 *μ*L of 0.2% (v/v) Triton X-100, 100 *μ*L of 25 *μ*g/mL PNPP. The 50 *μ*L of 3 mol/L NaOH was added to empty well as the blank control. The OD values were measured at 405 nm, and the OD per milligram of protein was calculated.

### 2.7. Statistical Analysis

Numerical data was analyzed using standard ANOVA techniques, and statistical difference was considered at *p* < 0.05. All the experiments were completed 5 times, with 3 replications used for each experiment.

## 3. Results and Discussion

### 3.1. Characterization of the Coatings

**Figure 2:**
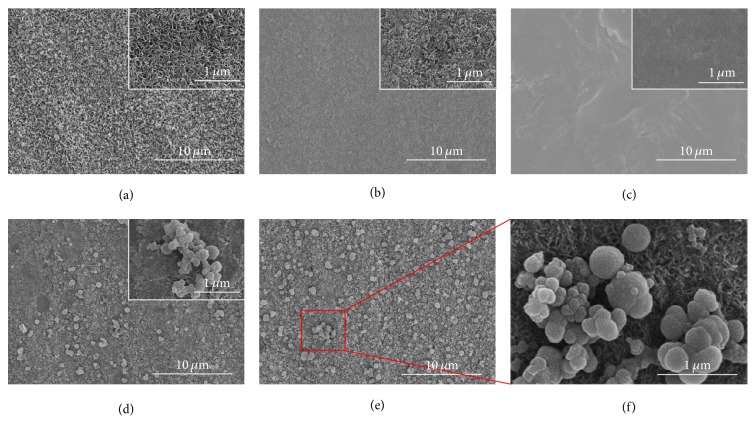
SEM images of the coatings: (a) Ti, (b) Ti/DOPA, (c) Ti/DOPA/SA, (d) Ti/DOPA/SA/Sr1, (e) Ti/DOPA/SA/Sr5, and (f) partially enlarged view of Ti/DOPA/SA/Sr5 (scale bars: 10 *μ*m).

Figure 2 shows the surface morphologies of the synthesized samples. After acid and alkali treatment (Figure 2(a)), Ti had a surface with a dense and porous structure, which is beneficial for covalent bond grafting [31]. On Ti/DOPA (Figure 2(b)), it can be clearly observed that the dense and porous structure was covered. The results demonstrated that DOPA successfully bonded in the Ti surface and the poly(dopamine) formed a distinctive layer [32]. On Ti/DOPA/SA (Figure 2(c)), the surface appears flat, which may be attributed to the fluidity of the SA [23]. Figures 2(d) and 2(e) show that abundant spherical particles with a uniform distribution on the surface are formed. A similar morphology has been previously shown by Li et al. [33]. Many hydroxyl and carboxyl compounds in SA have the chelation of Sr^2+^; meanwhile, Na^+^ can be substituted for Sr^2+^ to form strontium alginate, which presents in the form of a precipitate. Moreover, more spherical particles on Ti/DOPA/SA/Sr5 can be observed when compared with Ti/DOPA/SA/Sr1; this also demonstrates that SA has good chelation of Sr.

**Figure 3:**
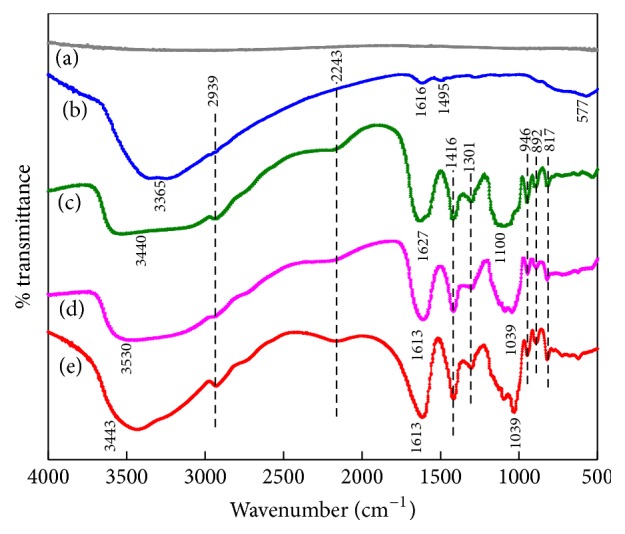
FT-IR spectra of the coatings: (a) Ti, (b) Ti/DOPA, (c) Ti/DOPA/SA, (d) Ti/DOPA/SA/Sr1, and (e) Ti/DOPA/SA/Sr5.

Figure 3 shows the FT-IR spectra of the synthesized samples in the range of 4000−500 cm^−1^. The spectra of Ti (Figure 3(a)) look smooth except the peak emerging at 568 cm^−1^, which is ascribed to the Ti-O-Ti stretching mode of TiO_2_ [34, 35]. The main characteristic peaks of Ti/DOPA (Figure 3(b)) are at 3365 cm^−1^ (-NH_2_ stretching), 2939 cm^−1^ (-CH_2_ stretching), 1616 cm^−1^ (-C=O stretching), 1495 cm^−1^ (-CH_2_ bending), and 577 cm^−1^ (Ti-O-Ti stretching) [36]. These emerging peaks indicate that DOPA is successfully coated on Ti. As shown in Figure 3(c), the alginate characteristic peaks of -OH stretching, -CH stretching, -NH stretching, -C=O stretching, -CH bending, and -C-O-C stretching are at 3530 cm^−1^, 2939 cm^−1^, 2243 cm^−1^, 1627 cm^−1^, 1416 cm^−1^, and 1100 cm^−1^, respectively [23, 37]. Among these peaks, 2243 cm^−1^ (-NH stretching) is attributed to the acid amide bond that is produced through the reaction of the amino group in DOPA and the hydroxyl in SA, and the result demonstrates that the coating of SA is successfully prepared. As shown in Figures 3(d) and 3(e), the alginate characteristic peaks show little differences except that a part of the peaks shifted to a lower wavenumber, which is attributed to the polar molecule SrCl_2_ enhancing the vibrational frequency of the polar bonds (-NH, >C=O) [38].

**Figure 4:**
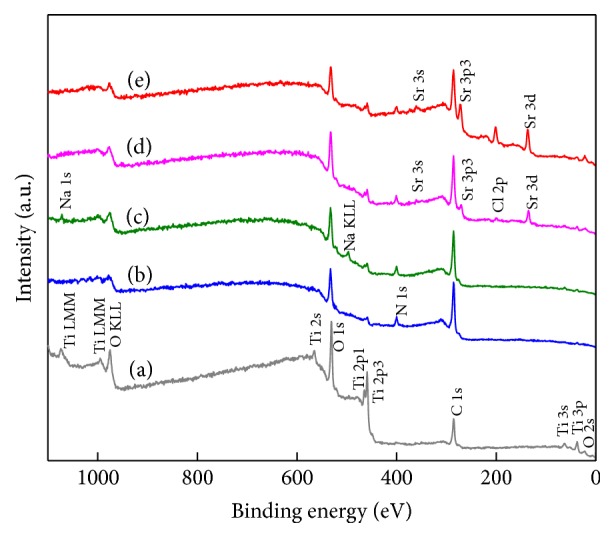
XPS spectra of the coatings: (a) Ti, (b) Ti/DOPA, (c) Ti/DOPA/SA, (d) Ti/DOPA/SA/Sr1, and (e) Ti/DOPA/SA/Sr5.

The chemical compositions of the surface after a series of modifications are determined by XPS. The XPS spectra of the synthesized samples and their corresponding surface elemental compositions are shown in Figure 4 and Table 1. On pure Ti (Figure 4(a)), the predominate components are C 1s, Ti 2p, and O 1s, and the binding energies of these peaks appear at 285 eV, 460 eV, and 531 eV, respectively. Carbon is typically present in the wide-scan spectrum of pure Ti due to the unavoidable hydrocarbon contamination, and it is used as an internal reference at 284.6 eV for calibrating peak positions [39]. On Ti/DOPA (Figure 4(b)), N 1s obviously emerges at 399 eV for the amino group in the DOPA. As shown in Table 1, the increase in the N and C contents and the decrease in the Ti content indicate the successful anchoring of dopamine on Ti. The carbon-to-nitrogen (C/N) ratio (10.44) for Ti/DOPA is higher than the dopamine (8.0), which is probably attributed to the surface carbon contamination of Ti. The increase in C 1s and O 1s signals coupled with the decrease in the Ti 2p signal is further accentuated on Ti/DOPA/SA (Figure 4(c)), indicating the success of the grafting process. On Ti/DOPA/SA/Sr1 (Figure 4(d)), the predominant peaks of Sr appear at 135 eV (Sr 3d), 270 eV (Sr 3p3), and 360 eV (Sr 3s), and the content of Sr is 2.6% (Table 1). It proves the success of the preparation of the Sr coating via chelating with SA. The peak of Sr is apparent, and the Sr content achieves 5.5% on Ti/DOPA/SA/Sr5. Therefore, the concentration of Sr in the Sr coating will increase accordingly with the improvement of Sr^2+^ concentration within 5 wt.%. This suggests that SA has good chelation of Sr when the Sr concentration is relatively low.

**Figure 5:**
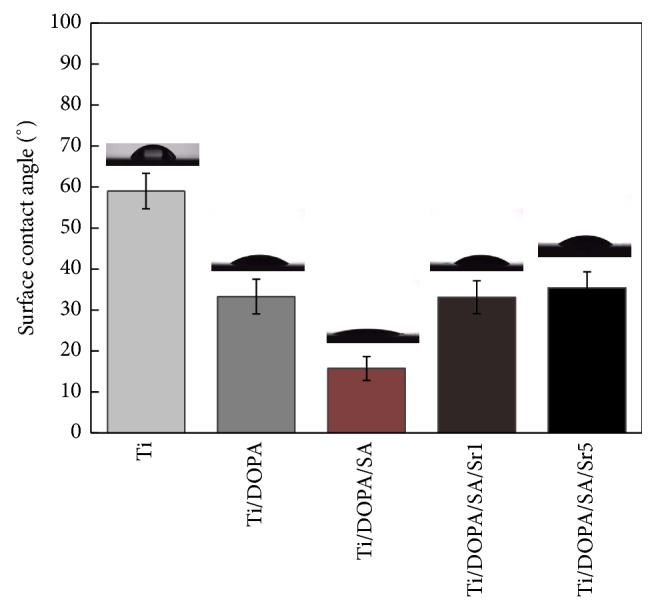
Water contact angles measured on various coatings.

Figure 5 shows the contact angle of the synthesized samples. The water contact angles on Ti/DOPA, Ti/DOPA/SA, Ti/DOPA/SA/Sr1, and Ti/DOPA/SA/Sr5 are 33.26° ± 4.35°, 15.71 ± 4.12°, 33.08° ± 3.65°, and 35.40° ± 1.68°, respectively, which are significantly smaller than that on pure Ti (59.00° ± 6.12°). This suggests that the surface modification can effectively improve the surface hydrophilicity of Ti. The hydrophilic quality of Ti/DOPA/SA, with the smallest contact angle among the synthesized samples, is obviously superior to the other coatings. This is because much of the hydroxyl presents as hydrophilic in the SA. When compared with Ti/DOPA/SA, the contact angles of Ti/DOPA/SA/Sr1 and Ti/DOPA/SA/Sr5 demonstrate a significant increase, and this suggests that the coating of Sr is successfully prepared. Water contact angle measurement can also provide supporting evidence that the Ti surface has been successfully modified.

### 3.2. Sr^2+^ Release Studies

**Figure 6:**
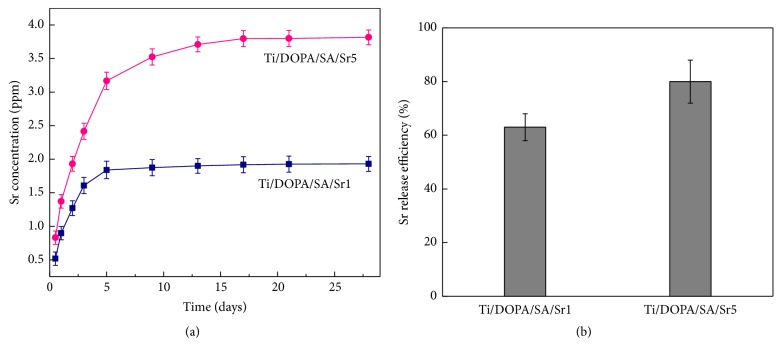
(a) Cumulative Sr^2+^ concentration of Ti/DOPA/SA/Sr1 and Ti/DOPA/SA/Sr5 as a function of time; (b) the Sr^2+^ release efficiency of Ti/DOPA/SA/Sr1 and Ti/DOPA/SA/Sr5 as a function of time.

The Sr^2+^ release behavior was assessed by immersing Ti/DOPA/SA/Sr1 and Ti/DOPA/SA/Sr5 in 5 mL PBS for up to 28 days. The Sr^2+^ release characteristics of Ti/DOPA/SA/Sr1 and Ti/DOPA/SA/Sr5 are presented in Figure 6. The release behavior can be described in 2 stages: a rapid burst and a slow release. In Figure 6(a), Sr^2+^ provides rapid burst from the alginate gel over the first 5 days. The average Sr^2+^ amounts released from Ti/DOPA/SA/Sr1 and Ti/DOPA/SA/Sr5 are 1.839 ppm and 3.168 ppm, respectively. The rapid burst may be attributed to the electrostatic deposition of a part of Sr^2+^ [2]. A stage of slow release is followed by rapid burst, and Sr^2+^ release gradually diminishes over 5−28 days. The average Sr^2+^ concentration released from Ti/DOPA/SA/Sr1 and Ti/DOPA/SA/Sr5 was reached at 1.930 ppm and 3.817 ppm, respectively, on the 28th day. The slow release of Sr is mainly due to the part of Sr^2+^ existing in the form of chelation with sodium alginate, and the covalent bond generated by chelation also plays an important role in the slow release.

At the early stage of implantation, the bone graft materials show rapid burst, which is beneficial in increasing the concentration of Sr^2+^ in the local area of the bone graft materials and makes them exhibit effective osteogenic activity. With the prolongation of soaking time, Sr^2+^ shows a slow release. The sustained release up to 1 month could promote Sr^2+^ in demonstrating osteogenic activity with long-term effectiveness in vivo [22, 40]. The sodium alginate chelating with Sr^2+^ shows a considerably extended overall Sr release, confirming the ability of this approach to achieve a long and sustained Sr release, which may be required for effective bone therapy.

### 3.3. Loading Efficiency of Sr^2+^

After the Sr release assay, the loading efficiency of Sr was evaluated. The loading efficiency was calculated from the formula *η* = *m*_1_/(*m*_1_ + *m*_*r*_), where *η* is the loading efficiency, *m*_1_ is the amount of the total release of Sr, and *m*_*r*_ is the amount of Sr in the rinse solution [41, 42].  Figure 6(b) shows the loading efficiency of Ti/DOPA/SA/Sr1 and Ti/DOPA/SA/Sr5. The results indicate that approximately 60%−80% of Sr was retained on the surface of Ti/DOPA/SA/Sr1 and Ti/DOPA/SA/Sr5 after an initial wash.

### 3.4. Cell Morphology

**Figure 7:**
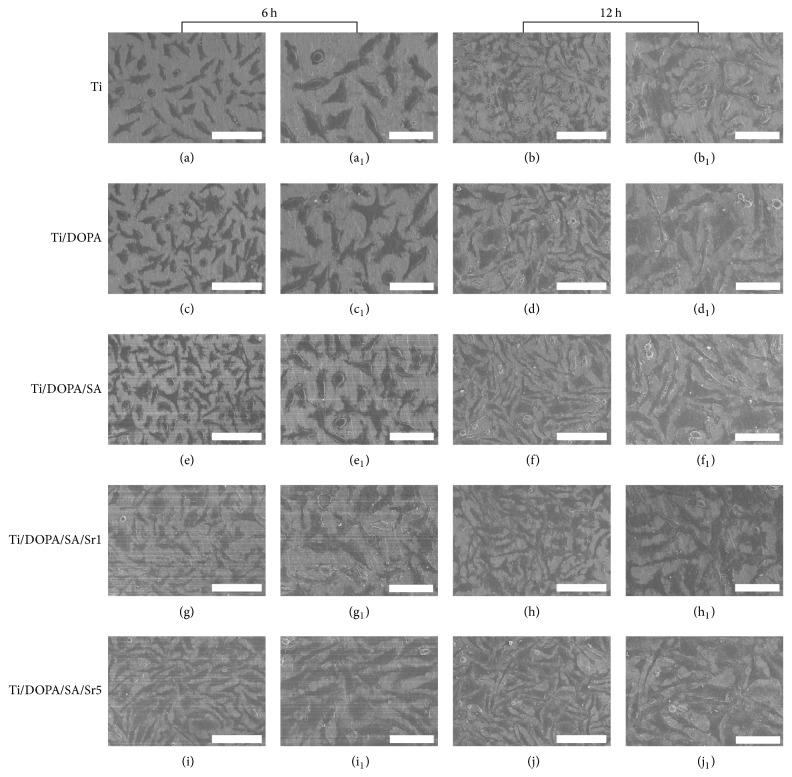
SEM images of cells after 6 h and 12 h of culture on different samples: (a, b) Ti, (c, d) Ti/DOPA, (e, f) Ti/DOPA/SA, (g, h) Ti/DOPA/SA/Sr1, and (i, k) Ti/DOPA/SA/Sr5 (scale bars: 100 *μ*m); (a_1_, b_1_) Ti, (c_1_, d_1_) Ti/DOPA, (e_1_, f_1_) Ti/DOPA/SA, (g_1_, h_1_) Ti/DOPA/SA/Sr1, and (i_1_, k_1_) Ti/DOPA/SA/Sr5 (scale bars: 50 *μ*m).

Cellular activities that demonstrate the enhancement of MG63 cell functions consist of cell spreading, proliferation, increased enzyme production (ALP), and mineralization (calcium deposition) [43, 44]. The attachment of MG63 cells to Ti is a prerequisite for the further cellular functions that lead to successful osseointegration of the implant in vivo [45].  Figure 7 shows the MG63 cells on various samples after seeding for 6 h and 12 h. On pure Ti (Figures 7(a) and 7(a_1_)), most of the MG63 cells remain as isolated and rounded single cells at 6 h. The behavior of the MG63 cells on Ti/DOPA (Figures 7(c) and 7(c_1_)) and Ti/DOPA/SA (Figures 7(e) and 7(e_1_)) is similar to that observed on pure Ti. On Ti/DOPA/SA/Sr1 (Figures 7(g) and 7(g_1_)) and Ti/DOPA/SA/Sr5 (Figures 7(i) and 7(i_1_)), however, dramatic difference can be observed, in that almost each cell spreads and links up to other cells. Panzavolta et al. [46] showed that Sr^2+^ incorporation has a slightly positive effect on early cellular attachment. This result is consistent with our findings. After 12 h cultivation (Figures 7(b) and 7(b_1_)), the MG63 cells did not spread completely on pure Ti. After DOPA and SA functionalization (Figures 7(d)-7(d_1_) and Figures 7(f)-7(f_1_)), it can be seen that the spreading of MG63 cells increased. On Ti/DOPA/SA/Sr1 and Ti/DOPA/SA/Sr5 (Figures 7(h)-7(h_1_) and Figures 7(k)-7(k_1_)), the number of attached cells was significantly higher. When compared with Ti/DOPA/SA/Sr1, further improvement of the cell number on Ti/DOPA/SA/Sr5 is attributed to Sr^2+^ incorporation. The current study definitively confirms the robust effect of Sr^2+^ incorporation on the adhesion and spreading of MG63 cells.

### 3.5. In Vitro Cytotoxicity

**Figure 8:**
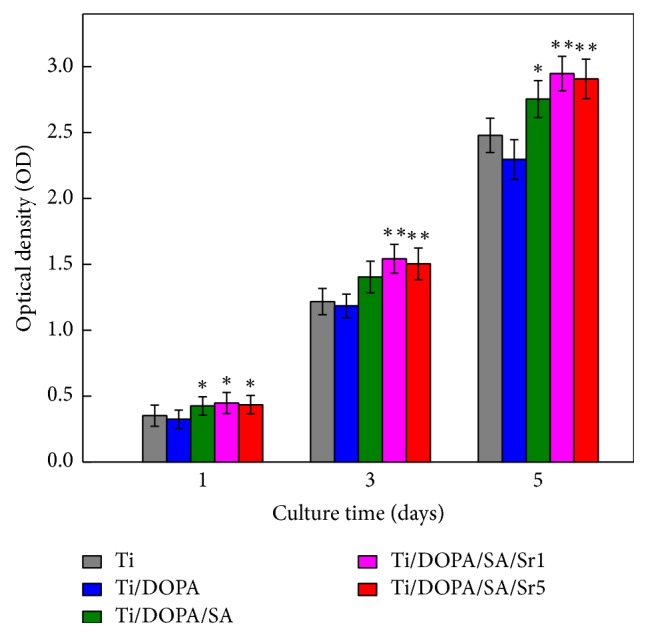
Cell viability of MG63 cells cultured for different periods of time on Ti, Ti/DOPA, Ti/DOPA/SA, Ti/DOPA/SA/Sr1, and Ti/DOPA/SA/Sr5 (^*∗*^*p* < 0.05 compared with Ti; ^*∗∗*^*p* < 0.01 compared with Ti).

Cell viability was evaluated by the performed MTT assay.  Figure 8 shows the MTT test result with the culture time of 1 d, 3 d, and 5 d at a density of 4 × 10^4^ cells/well in a 12-well plate. As shown in Figure 8, the cell viabilities on Ti and Ti/DOPA are comparatively low. Higher proliferation on Ti/DOPA/SA than that on Ti can be seen, which may be attributed to the excellent biocompatibility of SA [47]. It is generally believed that a large enough quantity of hydroxyl in SA could increase MG63 cell proliferation [48]. Both Ti/DOPA/SA/Sr1 and Ti/DOPA/SA/Sr5 showed comparatively more enhancement compared to that on Ti. The results obtained in the current study demonstrate that SA incorporated with Sr^2+^ could be more biocompatible.

### 3.6. Cell Adhesion, Proliferation, and Alkaline Phosphatase Activity

**Figure 9:**
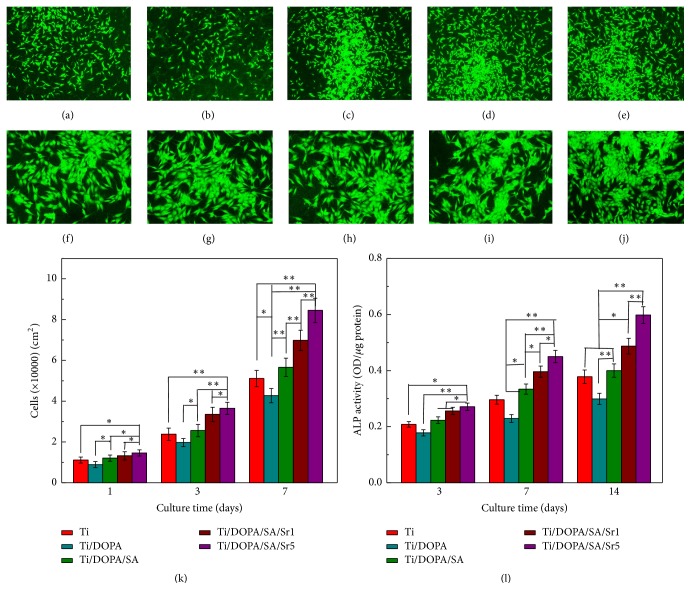
MG63 cells adhesion and distribution after 1 day of incubation. (a, f) Ti, (b, g) Ti/DOPA, (c, h) Ti/DOPA/SA, (d, i) Ti/DOPA/SA/Sr1, and (e, j) Ti/DOPA/SA/Sr5 ((a–e: magnified 40 times); (f–j: magnified 100 times)). (k) Cell proliferation measured by counting under a fluorescence microscope after 1, 3, and 7 days of incubation. (l) ALP activity of MG63 cells after 3, 7, and 14 days of incubation. Statistically significant differences (^*∗*^*p* < 0.05 and ^*∗∗*^*p* < 0.01).

Representative fluorescence microscopy images of the MG63 cells after 24 h cultivation were investigated to analyze the cell growth states on different samples. As shown in Figures 9(a)–9(j), the MG63 cells grew well on different samples. When compared with pure Ti (Figure 9(a)), Ti/DOPA (Figure 9(b)) has a relatively lower cell density. With the attached Sr^2+^ (Figures 9(e) and 9(f)), it can be seen that the spreading and the density of MG63 cells significantly increased. The results are consistent with the SEM.

The effect of surface functionalization on the proliferation of MG63 cells at days 1, 3, and 7 is shown in Figure 9(k). When compared with pure Ti, Ti/DOPA had a slight trend towards decreased proliferation and Ti/DOPA/SA showed no significant enhancement. However, on Ti/DOPA/SA/Sr1 and Ti/DOPA/SA/Sr5, significantly greater enhancement was exhibited on the proliferation than in the samples without Sr. With an increasing concentration of Sr^2+^, continuous enhancement can be observed. It indicates that the larger amount of released Sr^2+^ is in the safe dosage range [49].

ALP activity, which is widely used as a marker for early differentiation of osteoblast-like cells, is an important consideration for enhanced osseointegration besides the initial cell adhesion and proliferation [42, 50]. ALP activity was measured after the cells were cultured for 3, 7, and 14 days on different samples. As shown in Figure 9(l), ALP activities of MG63 cells cultured for 3 days on Ti, Ti/DOPA, and Ti/DOPA/SA were at a similar level. Both the ALP activities on Ti/DOPA/SA/Sr1 and Ti/DOPA/SA/Sr5 presented a slightly increased trend. When cultured for 7 days, the ALP activities of MG63 cells cultured on Ti/DOPA/SA/Sr1 and Ti/DOPA/SA/Sr5 presented significantly higher improvement than that on Ti. More significant improvement can be seen when MG63 cells were cultured for 14 days. A previous study showed that Sr can directly interact with the Ca-sensing receptor to enter into osteoblast cells and trigger mitogenic signals [51]. Figure 9(l) supports the notion that the Sr can stimulate MG63 cell differentiation and enhance the ALP activity.

## 4. Conclusion

A Sr-incorporated coating on a Ti surface was successfully fabricated by electrostatic immobilization and chemical bond grafting. With its excellent biocompatibility and stability, SA was selected as the suitable stabilizing agent to realize the uniform distribution of Sr. Long-lasting and controllable Sr release was observed on the Ti/DOPA/SA/Sr1 and Ti/DOPA/SA/Sr5 samples. Sr incorporation showed effectiveness on the adhesion, cytotoxicity, proliferation, and ALP activity. A higher Sr content resulted in more effective functioning in terms of promoting the osteogenic activity of MG63 cells. Such Sr-incorporated coatings could provide a promising solution for promoting the tissue integration of implants; thus, they have considerable potential for use as bone substitutes in orthopedic applications.

## Figures and Tables

**Table 1:** Elemental composition at the surface of pristine and functionalized Ti substrates as determined by XPS.

Substrate	C%	O%	Ti%	N%	Sr%
Ti	42.6 ± 0.46	41.8 ± 0.57	15.6 ± 0.74	—	—
Ti/DOPA	63.7 ± 0.58	21.5 ± 0.49	8.7 ± 0.68	6.1 ± 0.56	—
Ti/DOPA/SA	66.5 ± 0.23	24.7 ± 0.28	2.0 ± 0.78	6.8 ± 0.50	—
Ti/DOPA/SA/Sr1	66.0 ± 0.48	24.5 ± 0.72	2.1 ± 0.38	4.8 ± 0.39	2.6 ± 0.82
Ti/DOPA/SA/Sr5	64.6 ± 0.39	23.6 ± 0.61	1.7 ± 0.45	4.6 ± 0.51	5.5 ± 0.76
